# Diagnostic accuracy of autoverification and guidance system for COVID-19 RT-PCR results

**DOI:** 10.1007/s13167-022-00310-4

**Published:** 2022-12-16

**Authors:** Yingmu Cai, Mengyu Liu, Zhiyuan Wu, Cuihong Tian, Song Qiu, Zhen Li, Feng Xu, Wei Li, Yan Zheng, Aijuan Xu, Longxu Xie, Xuerui Tan

**Affiliations:** 1 Joint Laboratory of Shantou University Medical College and Guangdong Hybribio Biotech Ltd, Shantou University Medical College, Shantou, 515041 Guangdong China; 2 Hybribio Medical Laboratory Group Ltd, Chaozhou, 521000 Guangdong China; 3 Clinical Research Centre, The First Affiliated Hospital of Shantou University Medical College, Shantou, 515041 Guangdong China; 4 Beijing Municipal Key Laboratory of Clinical Epidemiology, School of Public Health, Capital Medical University, Beijing, 100069 China; 5 Centre for Precision Health, Edith Cowan University, Perth, WA 6027 Australia; 6 Department of Cardiovascular Medicine, The First Affiliated Hospital of Shantou University Medical College, Shantou, 515041 Guangdong China; 7 Human Papillomavirus Molecular Diagnostic Engineering Technology Research Centre, Chaozhou, 521000 Guangdong China; 8 Department of Research and Development, Guangdong Research Institute of Genetic Diagnostic and Engineering Technologies for Thalassemia, Chaozhou, 521011 Guangdong China

**Keywords:** Predictive preventive personalized medicine (PPPM/3PM), COVID-19, Autoverification and guidance system (AGS), Reverse transcriptase-polymerase chain reaction (RT-PCR), Manual interpretation, Diagnostic accuracy, Prevention and control, Personalized policy, Dynamic COVID-free policy

## Abstract

**Background:**

To date, most countries worldwide have declared that the pandemic of COVID-19 is over, while the WHO has not officially ended the COVID-19 pandemic, and China still insists on the personalized dynamic COVID-free policy. Large-scale nucleic acid testing in Chinese communities and the manual interpretation for SARS-CoV-2 nucleic acid detection results pose a huge challenge for labour, quality and turnaround time (TAT) requirements. To solve this specific issue while increase the efficiency and accuracy of interpretation, we created an autoverification and guidance system (AGS) that can automatically interpret and report the COVID-19 reverse transcriptase-polymerase chain reaction (RT-PCR) results relaying on computer-based autoverification procedure and then validated its performance in real-world environments. This would be conductive to transmission risk prediction, COVID-19 prevention and control and timely medical treatment for positive patients in the context of the predictive, preventive and personalized medicine (PPPM).

**Methods:**

A diagnostic accuracy test was conducted with 380,693 participants from two COVID-19 test sites in China, the Hong Kong Hybribio Medical Laboratory (*n* = 266,035) and the mobile medical shelter at a Shanghai airport (*n* = 114,658). These participants underwent SARS-CoV-2 RT-PCR from March 28 to April 10, 2022. All RT-PCR results were interpreted by laboratorians and by using AGS simultaneously. Considering the manual interpretation as gold standard, the sensitivity, specificity, positive predictive value (PPV), negative predictive value (NPV) and accuracy were applied to evaluate the diagnostic value of the AGS on the interpretation of RT-PCR results.

**Results:**

Among the 266,035 samples in Hong Kong, there were 16,356 (6.15%) positive, 231,073 (86.86%) negative, 18,606 (6.99%) indefinite, 231,073 (86.86%, negative) no retest required and 34,962 (13.14%, positive and indefinite) retest required; the 114,658 samples in Shanghai consisted of 76 (0.07%) positive, 109,956 (95.90%) negative, 4626 (4.03%) indefinite, 109,956 (95.90%, negative) no retest required and 4702 (4.10%, positive and indefinite) retest required. Compared to the fashioned manual interpretation, the AGS is a procedure of high accuracy [99.96% (95%CI, 99.95–99.97%) in Hong Kong and 100% (95%CI, 100–100%) in Shanghai] with perfect sensitivity [99.98% (95%CI, 99.97–99.98%) in Hong Kong and 100% (95%CI, 100–100%) in Shanghai], specificity [99.87% (95%CI, 99.82–99.90%) in Hong Kong and 100% (95%CI, 99.92–100%) in Shanghai], PPV [99.98% (95%CI, 99.97–99.99%) in Hong Kong and 100% (95%CI, 99.99–100%) in Shanghai] and NPV [99.85% (95%CI, 99.80–99.88%) in Hong Kong and 100% (95%CI, 99.90–100%) in Shanghai]. The need for manual interpretation of total samples was dramatically reduced from 100% to 13.1% and the interpretation time fell from 53 h to 26 min in Hong Kong; while the manual interpretation of total samples was decreased from 100% to 4.1% and the interpretation time dropped from 20 h to 16 min at Shanghai.

**Conclusions:**

The AGS is a procedure of high accuracy and significantly relieves both labour and time from the challenge of large-scale screening of SARS-CoV-2 using RT-PCR. It should be recommended as a powerful screening, diagnostic and predictive system for SARS-CoV-2 to contribute timely the ending of the COVID-19 pandemic following the concept of PPPM.

## Introduction

### The dynamic COVID-free policy applied by China

The coronavirus disease 2019 (COVID-19) has become the most widespread global pandemic since it was first reported on December 30, 2019 [1, 2], causing huge health and economic burden [3]. Nowadays, there is a trend to declare the ending of COVID-19 globally, while China is still pursuing the dynamic COVID-free strategy due to high population density and limited medical resources. Based on the susceptibility of COVID-19 [4], wearing a mask and presenting a negative nucleic acid testing result within 48 h are necessary when people enter public places such as airport, railway station and supermarket. Under this strict control and prevention measures, the dynamic COVID-free strategy applied by China contributes to the lowest infection and mortality rates, aligning with the concept of predictive, preventive and personalized medicine (PPPM) [5, 6].

### Challenges in preventing COVID-19

The diagnosis of COVID-19 is based on the comprehensive analysis of epidemiological history, clinical manifestation, laboratory and imaging examination [7]. Among them, nucleic acid amplification test (NAAT) of SARS-CoV-2 is critically important [8, 9], and the reverse transcriptase-polymerase chain reaction (RT-PCR) test is the gold standard for the identification of SARS-CoV-2 in the specimens collected from the upper and lower respiratory tracts [10, 11], playing a vital role in the pandemic secondary prevention and control with a PPPM approach [12].

Under the dynamic COVID-free policy in China, the number of daily samples sent to a laboratory at the peak time, a period when a mass nucleic acid screening with a 100% coverage of population for several days are conducted in the residential communities, was over 10 times higher than that of the routine time, a period when only one person entering airport or railway station or person working in the industry with high risk for COVID-19 are required to do the nucleic acid testing. It poses a huge challenge for the quality and efficiency of the interpretation of the RT-PCR results. The gigantic workload is far beyond the capacity of personnel audit and thus commonly causes a delay in data reporting. However, all laboratories globally conducting nucleic acid detection of SARS-CoV-2 rely on manual interpretation of RT-PCR results presently, which is labour intensive and time-consuming and sometime fails to meet the requirements of turnaround times (TATs). Manual interpretation of RT-PCR results also demands accredited laboratorian qualified for the standardized audit procedures after training. Both false negative and false positive reports are the major challenges caused by the inconsistency of individual result’s interpretation. For example, the false negative reports of RT-PCR can lead to a wrong management of the positive cases, thus resulting in the processive spread of the pandemic [13, 14]. Moreover, the lack of standardized guidance for indefinite or suspected positive cases may also lead to extended TATs. Therefore, a paradigm change from reactive medicine to PPPM, and a more prompt and accurate diagnosis of COVID-19 is of great significance in reducing treatment delay and preventing the epidemic from spread.

### An optimal autoverification to diagnose COVID-19 in the perspective of PPPM

Autoverification, based on a series of simple and complex algorithms, provides automated actions operated by a computer system to release the COVID-19 RT-PCR results. It has been shown that autoverification can reduce the bias from manual interpretation, improve the efficiency and accuracy of test results and enable laboratory technicians to pay more attention to some suspicious positive samples [15, 16]. Therefore, the popularization and application of autoverification under the personalized mass nucleic acid testing condition may be beneficial to predict the effective reproduction number at time (Rt), a measure reflecting the real-time transmissibility of an epidemic, providing strong evidence to diagnose, predict, prevent and control COVID-19 from the perspective of PPPM.

### Working hypothesis

To solve these issues faced by China and to prevent COVID-19 in the framework of PPPM, we developed an autoverification and guidance system (AGS), which is able to examine the preanalytical, analytical and postanalytical data based on a unified standardized criterion. It is hypothesized that the AGS may be a more effective approach with higher accuracy in diagnosis, prediction, prevention and control COVID-19 than that of conventional manual interpretation. Then, we tested and validated the accuracy of AGS in the interpretation of COVID-19 RT-PCR results based on the tests from the Hong Kong and Shanghai cohorts.

## Methods

### Clinical samples

The data of 266,035 nasopharyngeal samples from Hong Kong and 114,658 oropharyngeal samples from Shanghai during the period from March 28 to April 10, 2022, were collected for the evaluation of the AGS. Hong Kong was characterized by its high positive rate of the infection (12.1%, Table 3), while Shanghai has a low positive rate of the infection (0.01%, Table 3) and high requirement for timeliness but under low quality control environment. All the tests were performed at the Hong Kong Hybribio Medical Laboratory and the Hybribio mobile medical shelter at Shanghai airport. The data were stored in the Laboratory Information System, an information management hub serving for data collection, reporting, transmission and archiving.

### Design of the AGS

**Fig. 1:**
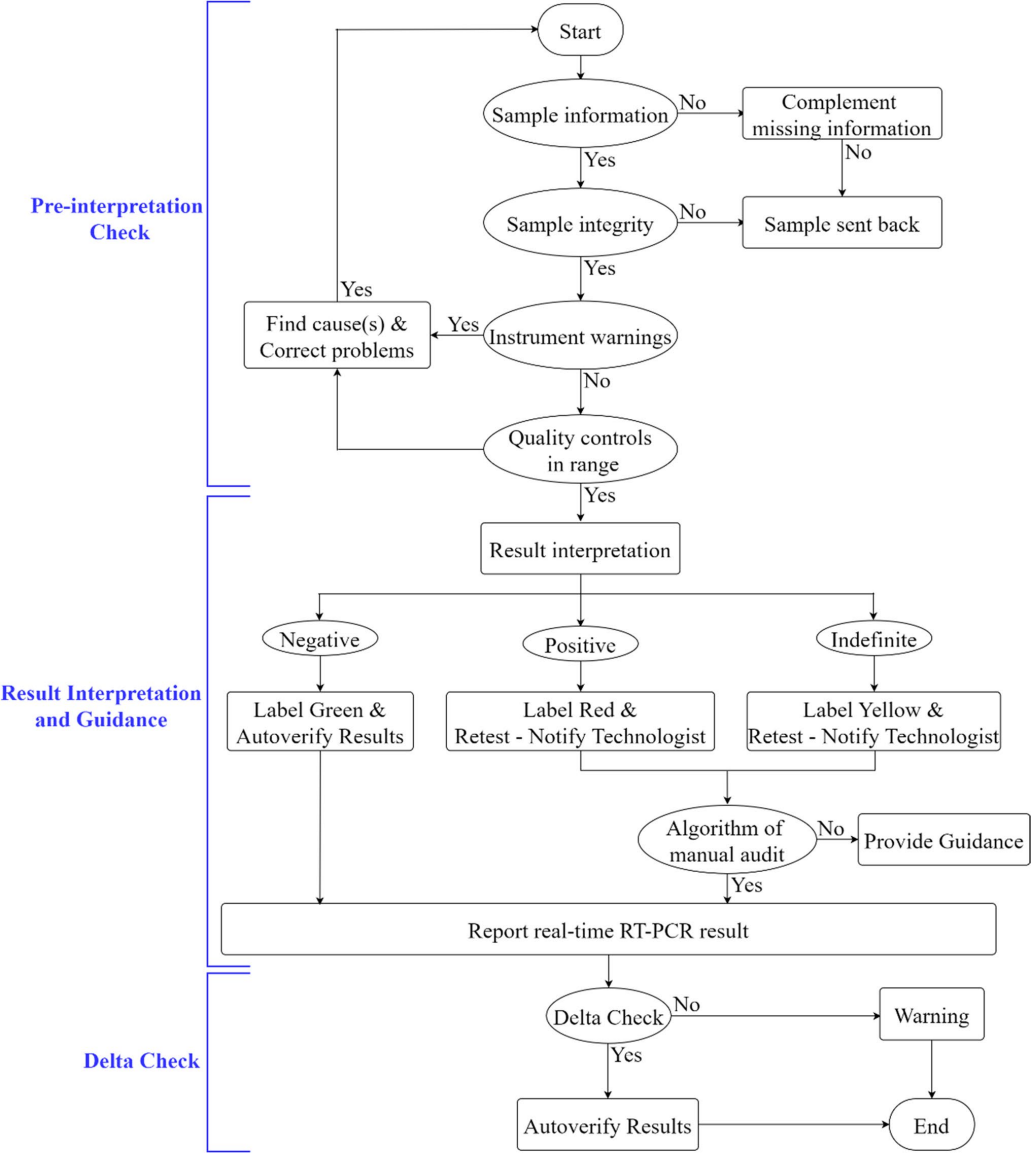
Flow diagram of the AGS. The AGS consists of three stages for each sample testing: pre-interpretation check, result interpretation and guidance, delta check. The recorded information of a specimen invalidating one of these rules is asked to complement the missing information and then run through the system again. Only the samples with integral information are processed; otherwise, the samples will be sent back for re-sampling and retesting. The results of the RT-PCR are classified into negative (labelled green), positive (labelled red) and indefinite (labelled yellow) after passing the rules in the pre-interpretation check stage. A negative sample is autoverified by the AGS and reported immediately. While the positive and indefinite cases require further manual interpretation and subsequent guidance. If the sample pass the delta check, the results will be autoverified; otherwise, the system will give a warning

The flow diagram (Fig. 1) of the AGS consists of three stages for each sample testing: (1) pre-interpretation check, (2) result interpretation and guidance and (3) delta check. The design of algorithms is based on the guideline from the Clinical and Laboratory Standards Institute (CLSI) [17, 18]. Once a RT-PCR reaction finishes, the data will be stored automatically in the Laboratory Information System and exported as a new file to AGS. Then, the file is processed through the following steps shown in the flowchart (Fig. 1). The system will generate a disposal warning when a sample invalidates any one of the rules, which are set based on the specimen information and integrity, and then report the exact issues that need to be fixed by manual intervention. If the problems cannot be solved by laboratory personnel intervention, the system will advise for re-sampling and retesting.

### Rules of specimen information and integrity

The recorded information of the subjects includes a unique sample identifier, a unique subject identifier, age and/or date of birth, gender, sample type, sample collection date and location. A specimen invalidating one of these rules is asked to complement the missing information and then run through the system again. Only the samples with integral information in the sealed preservation tubes are processed, otherwise re-sampling will be requested.

### Rules of instrument error flags or warnings

Once an instrument error flag warning appears, a manual intervention will be required to identify the cause(s). After the problem being fixed, the samples will run through the entire process again.

### Rules of quality control and internal control

Each RT-PCR test includes both negative and positive quality controls. In this study, the passed quality control algorithm and the cycle threshold (Ct) value of the internal control are in compliance with the instructions of COVID-19 RT-PCR detection kit and the policies of Hong Kong and Shanghai Centres for Disease Control [19]. After passing the rules of quality controls, the samples in the RT-PCR running will be analysed in the stage of result interpretation and guidance. The reference Ct value of the internal control for a human pharyngeal swab in this study is set between 15 and 36. Samples with the Ct value of the internal control out of this range will be classified as indefinite cases that require retest.

### Rules of result interpretation and guidance

The RT-PCR test targets at two SARS-CoV-2 genomic sequences, the open reading frame1ab (ORF1ab) and gene coding for nucleocapsid protein (N) [19]. The results of the RT-PCR are classified into negative (labelled green), positive (labelled red) and indefinite (labelled yellow) after passing the rules in the pre-interpretation check stage. The whole time of AGS or laboratorians spending on interpreting all samples collected during a specific period was considered as the interpretation time. Due to the gradually decreased fatality rate of COVID-19, a less rigid prevention and control policy is launched in China. Thus, in the present study, the reference Ct value of the positive is set between 10 and 35 for both ORF1ab and N. Assessment of test results for clinical specimens passing the rule of internal control is interpreted according to the following criteria:

(1) A specimen with sigmoidal amplification curves of both ORF1ab and N, both Ct values for ORF1ab and N between 10 and 35, and the discrepancy between the Ct values of the two targeted genes ≤ 3 is regarded as a positive case.

(2) A specimen with no sigmoidal amplification curves of both ORF1ab and N, and Ct values of the two targeted genes denoted as “Undet”, i.e. undetermined, in the software interface is considered as a negative case.

(3) Samples that cannot be classified as neither negative nor positive are defined as indefinite cases, including the specimens with abnormal curve(s) of ORF1ab and/or N, the specimens with the Ct value(s) of ORF1ab and/or N out of the range of 10–35, and the specimens with the discrepancy between the Ct values of the two targeted genes > 3. The possible reasons for indefinite results and the subsequent guidance after initial RT-PCR interpretation are listed in Table 1.

**Table 1:** Guidance for positive and indefinite RT-PCR results

Categories after initial RT-PCR			Possible reason(s)	Re-test results	Guidance
Positive			N/A	Negative	Report
				Positive	Report
Indefinite	Ct value of internal control out of the range of 15–36		Fail in sample collection, extraction, reverse transcription or mistake in reagent adding process	Out of the range	Resampling
				In the range	Guidance based on the classification of the re-test result
	Abnormal curve(s)		Instrument malfunction; interruption during RT-PCR reaction; interferent(s) in the RT-PCR solution; RT-PCR tubes not sealed	Abnormal curve(s)	Check the conditions of RT-PCR instrument and reagent. Resampling if both are in good function
				Normal curves	Guidance based on the classification of the re-test result
	Ct value(s) of the ORF1ab and/or N out of the range of 10–35	One gene’s Ct value out of the range	Low extraction efficiency due to the mismatch among preservation tubes, extraction instrument and solution; low copy number of virus in the sample	Negative	Report
				Positive	Report
				Same result as the initial RT-PCR	Label positive and report
		Both genes’ Ct values out of the range	Patient in recovery period; participants were vaccinated with the COVID-19 vaccine within 48 h	Negative	Report
				Positive	Report
				Same result as the initial RT-PCR	Clarify whether the participants were vaccinated within 48 h and resampling
	The discrepancy between the Ct values of the ORF1ab and N exceed 3		Fragmentary DNA after extraction and reverse transcription; discrepant amplification efficiency between ORF1ab and N genes	Negative	Report
				Positive	Report
				Same result as the initial RT-PCR	Label positive and report

*Ct*, cycle threshold; *N/A*, not available; *N*, gene coding for nucleocapsid protein; *ORF1ab*, open reading frame1ab

Samples interpreted as negative after initial RT-PCR were reported immediately, while positive and indefinite samples required re-test. During the re-test stage, if the RT-PCR results are inconsistent using more than 3 kinds of kits from different companies, the system will advise resampling. The guidance in this table is based on consistent result in re-test

### Rules of delta check

As the last process of autoverification system, the delta check refers to the comparation of a specific and predefined result against a subject’s previous laboratory result(s) [18]. Delta check in AGS includes the comparison with historical results of RT-PCR testing, and the results of further COVID-19 antigen or antibody testing, which are done within 1 week of RT-PCR testing. If the comparison fails to pass the delta check in the AGS algorithm, the system gives a warning, and then a detailed description is added in the final report. The algorithm for the comparison between the RT-PCR result and an antigen test/immunoglobulin M (IgM) test/immunoglobulin G (IgG) test is shown in the Table 2.

**Table 2:** Algorithms of comparison between RT-PCR and antigen or antibody tests

RT-PCR	Antigen test			IgM test			IgG test	
	Positive	Negative		Positive	Negative		Positive	Negative
Positive	Pass	Fail		Pass	Fail		Fail	Pass
Negative	Fail	Pass		Fail	Pass		Pass	Fail

*IgM*, immunoglobulin M; *IgG*, immunoglobulin G

### Statistical analysis

Analysis of the data was performed using the SPSS statistics software package, version 26.0 for Microsoft Windows (SPSS Inc., Chicago, IL, USA). The interpretation of the initial RT-PCR screening results was recognized as negative, positive and indefinite. Among them, the negative cases were reported immediately, while the positive and indefinite samples were further retested by senior technicians. In order to assess the performance of the system, the interpretation outcomes were classified into two categories: no retest required (negative) and retest required (positive + indefinite). Considering the manual interpretation for COVID-19 RT-PCR as the gold standard, the sensitivity, specificity, positive predictive value (PPV), negative predictive value (NPV), accuracy, 95% confidence interval (95%CI) and Cohen’s kappa of AGS were calculated using an online statistical tool [20].

## Results

To assess the performance of the AGS, the initial RT-PCR results of 380,693 samples (266,035 from Hong Kong; 114,658 from Shanghai) were processed by the AGS and senior technologists simultaneously (Table 3). Without support of the AGS, all RT-PCR results needed to be read by laboratorians, i.e. a 100% manual interpretation rate. While the AGS was applied, the application of manual interpretation was reduced to 13.1%, and the total interpretation time of all the samples in the initial RT-PCR screening fell from 53 h to 26 min in Hong Kong. In the case of the mobile medical shelter in Shanghai, the manual interpretation rate of all samples was cut down to 4.1%, and the total interpretation time dropped from 20 h to 16 min. These demonstrated the advantages of the AGS in reducing manpower input and TAT.

**Table 3:** Comparison between the AGS and manual auditing in live environments

			Hong Kong (*n* = 266,035)		Shanghai (*n* = 114,658)	
			Autoverification	Manual audit	Autoverification	Manual audit
Initial RT-PCR results	Total number of runs*		3315^a^	3315^a^	1547^b^	1547^b^
	Instrument error flags or warnings		0	0	0	0
	Quality controls	Pass	255,841	255,841	111,240	111,240
		Fail	10,194	10,194	3,418	3,418
	Number of autoverified cases		231,066	N/A	109,956	N/A
	Autoverification rate (%)		86.9%	N/A	95.9%	N/A
	Number of manual interpretations		34,969	266,035	4,702	114,658
	Manual interpretation rate (%)		13.1%	100%	4.1%	100%
	Total interpretation time*		26 min^c^	53 h^d^	16 min^e^	20 h^f^
Final reporting	Positive		32,198		12	
	Negative		232,218		114,035	
	Indefinite		1,619		611	
	Positive rate		12.1%		0.01%	

*N/A*, not available

^*^Total number of runs: the RT-PCR rounds of samples experienced

^a^Total 3315 rounds RT-PCR with maximum 90 samples per round were conducted to finish the 226,035 samples testing in Hong Kong

^b^Total 1547 rounds RT-PCR with about 75 samples per round were conducted to finish the 114,658 samples testing in Shanghai

^*^Total interpretation time: the time spending on the interpretation of all samples

^**c**^The time spending on interpreting 266,035 samples by the AGS

^d^The time spending on interpreting 266,035 samples by laboratorians

^e^The time spending on interpreting 114,658 samples by the AGS

^f^The time spending on interpreting 114,658 samples by laboratorians

The interpreted outcomes of the initial RT-PCR tests were classified into positive, negative and indefinite, among which positive and indefinite samples were further retested by senior technicians while the negative cases were reported immediately. Of the 266,035 nasopharyngeal samples in Hong Kong by manual audit, 16,356 (6.15%) were positive, 231,073 (86.86%) were negative, and 18,606 (6.99%) were indefinite; 231,073 (86.86%, negative) did not require to be further retested, and 34,962 (13.14%, positive and indefinite) required to be further retested; of the 114,658 oropharyngeal samples in Shanghai, 76 (0.07%) were positive, 109,956 (95.90%) were negative, and 4,626 (4.03%) were indefinite; 109,956 (95.90%, negative) did not require to be further retested, and 4702 (4.10%, positive and indefinite) required to be further retested. The comparison of results interpretation between the AGS and manual audit is listed in Table 4. The interpretation outcome was defined into “no retest required group” and “retest required group” as shown in Table 5. The AGS showed 99.98% (95%CI, 99.97–99.98%) sensitivity, 99.87% (95%CI, 99.82–99.90%) specificity, 99.98% (95%CI, 99.97–99.99%) PPV, 99.85% (95%CI, 99.80–99.88%) NPV and 99.96% (95%CI, 99.95–99.97%) accuracy, with a 0.998 Cohen’s kappa in Hong Kong; while 100% (95%CI, 100–100%) sensitivity, 100% (95%CI, 99.92–100%) specificity, 100% (95%CI, 99.99–100%) PPV, 100% (95%CI, 99.90–100%) NPV, and 100% (95%CI, 100–100%) accuracy, with a Cohen’s kappa of 1.000 at Shanghai and a *P* value under 0.001 (Table 6), suggesting a perfect accuracy of the AGS in the real-world application.

**Table 4:** Autoverification and manual audit results of the initial RT-PCR screening, classified as positive, negative, and indefinite

Autoverification results of the initial RT-PCR		Manual audit results of the initial RT-PCR			Total
		Positive	Negative	Indefinite	
Hong Kong (Accuracy: 99.93%)	Positive	16,323	0	59	16,382
	Negative	0	231,020	46	231,066
	Indefinite	33	53	18,501	18,554
	Total	16,356	231,073	18,606	266,035
Shanghai (Accuracy: 99.98%)	Positive	76	0	23	99
	Negative	0	109,956	0	109,956
	Indefinite	0	0	4603	4603
	Total	76	109,956	4626	114,658

**Table 5:** Autoverification and manual audit results of the initial RT-PCR screening classified as no retest required and retest required

Autoverification results of initial RT-PCR		Manual audit results of initial RT-PCR		Total
		No retest required^a^	Retest required^b^	
Hong Kong	No retest required^a^	231,020	46	231,066
	Retest required^b^	53	34,916	34,969
	Total	231,073	34,962	266,035
Shanghai	No retest required^a^	109,956	0	109,956
	Retest required^b^	0	4,702	4,702
	Total	109,956	4,702	114,658

^a^No retest required (negative)

^**b**^Retest required (positive + indefinite)

**Table 6:** Evaluation the performance of the AGS

	Hong Kong	Shanghai
Sensitivity	99.98% (95%CI, 99.97–99.98%)	100% (95%CI, 100–100%)
Specificity	99.87% (95%CI, 99.82–99.90%)	100% (95%CI, 99.92–100%)
Youden index	0.9985	1
PPV	99.98% (95%CI, 99.97–99.99%)	100% (95%CI, 99.99–100%)
NPV	99.85% (95%CI, 99.80–99.88%)	100% (95%CI, 99.90–100%)
Accuracy	99.96% (95%CI, 99.95–99.97%)	100% (95%CI, 100–100%)
Cohen’s kappa	0.998	1
*P* value	< 0.001	< 0.001

*PPV*, positive predictive value; *NPV*, negative predictive value; *95%CI*, 95% confidence interval

Calculated sensitivity, specificity, Youden index, PPV, NPV, accuracy, Cohen’s kappa, and *P* value of the AGS implemented in Hong Kong and Shanghai

## Discussion

RT-PCR is a critical tool in fighting against SARS-CoV-2, being the gold standard for the identification of the virus in upper and lower respiratory tract specimens [10, 11]. Identifying infected patients and separating them from uninfected people are the most efficient approaches to prevent and control the spread of SARS-CoV-2. Under the condition of mass nucleic acid screening in China, a timely, accurate and consistent RT-PCR test and subsequent accurate interpretation of the results are the basic foundation for infection diagnosis, prevention and management in a real-world setting following the concept of PPPM.

Previous studies showed that autoverification has outstanding performance in processing the multiple types of laboratory data, e.g. thalassemia gene detection [21], arterial blood gas analysis [22] and enzyme-linked immunosorbent assay (ELISA) of hepatitis B virus (HBV) serological markers [23]. The implementation of autoverification exhibits advantages in reducing error rate, saving time, decreasing manpower demand and simplifying the processing of sophisticated manual auditing [18]. In this study, the AGS we created to identify potential analytical errors and replace manual interpretation procedure shows great performance in time-efficient, labour-saving and diagnosis-precisive, being a good example of PPPM practice in pandemic control.

The asymptomatic raised greatly and only a few were transformed to confirmed cases due to the declined virulence of COVID-19, vaccine development and rapid screening [24–27]. However, China still adopts strict pandemic prevention and control. It is “a personalized issue” under “a personalized policy” at communities level, following the “dynamic COVID-free strategy” in China. Therefore, the AGS with high accuracy and efficacy is warranted to solve this specific challenge in China. This study is the first attempt to apply and assess the autoverification in Chinese large-scale COVID-19 screenings during the pandemic. The AGS conducted in this study provides a platform to rapidly distinguish negative results from the outcomes that need manual interpretation, being suitable for the dynamic COVID-free policy in China and beneficial to the secondary prevention and management of COVID-19 in PPPM approach. The computer-based immediate verification and report of negative results save a large amount of time on reporting negative cases when it compares to the manual audit mode and thus enable laboratorians to pay more attention to the cases that require retest in terms of personalized accuracy at large communities level screening which is a common challenge in PPPM practice.

The AGS is linked to the Laboratory Information System to ensure the confidentiality of data, and the computer-based autoverification algorithm guarantees excellent accuracy (99.98% in Hong Kong and 100% in Shanghai) by avoiding human errors. In the case of the results that require manual interpretation, subsequent guidance is provided by the AGS to the laboratorians. In addition, the implementation of AGS system is especially important for countries and regions with low resource settings where the expertise for manual interpretation of RT-PCR results is inadequate.

The pre-interpretation check of the AGS is implemented to warn errors during in-lab workflow, like instrument breakdown and contamination of reagents. While the process of delta check is used to further validate the accuracy of AGS by comparing the RT-PCR results with antigen or antibody testing results. Both NAAT and antigen test are intended for the detection of current infection. In contrast to NAAT that detects the existence of viral nucleic acid, the antigen test detects the nucleocapsid protein antigen of the SARS-CoV-2 in nasal swabs, nasopharyngeal swabs or saliva [8, 28]. However, the antigen test always has lower sensitivity and specificity compared to the RT-PCR test, especially in samples with low virus load [10, 11, 29]. An antibody test is intended for the detection of SARS-CoV-2 antibodies (IgG and/or IgM) in blood which can help determine a past infection [2, 8], but it may also generate false positive results for people who have received a SARS-CoV-2 vaccine [30]. Therefore, the failure of samples passing the delta check is mainly due to the samples taken from infected people in latent period and/or due to the low sensitivity of antigen or antibody tests. The diagnostic accuracy of RT-PCR of SARS-CoV-2 is further increased by retesting the samples that cannot pass the algorithm of delta check.

The operation performance of the AGS was tested in two different scenarios: the Hong Kong medical laboratory and the mobile medical shelter at Shanghai airport. The positive rate in Hong Kong was 12.1%, much higher when compared to 0.01% in Shanghai. The mobile medical shelter is usually constructed in place where is not suitable for building a permanent medical laboratory. Therefore, it is a big challenge to provide quality control on data analysis in a mobile shelter where has a high risk of low quality of sample handling and data processing. Under such conditions, the AGS was demonstrated to be a powerful tool by ensuring standardized data process with autoverification. It also showed perfect accuracy of interpretation and had considerable time efficiency to save the labour resource, meeting the testing demanding a PPPM approach in the situation of COVID-19 pandemic.

The twenty-first century witnessed the development of medical services from traditional, complementary and alternative medicine (TCAM), person-centred medicine (PCM), individualized medicine (IM), stratified medicine (SM), personalized medicine (PM) and precision medicine to PPPM [31]. PPPM combines the advantages and minimizes the disadvantages of the existing approaches and has been considered as the “medicine of the future” [5]. It has been successfully applied to non-communicable diseases including cancer [32, 33], cardiovascular diseases [34], ischemic stroke [35] and neurodegenerative diseases [36] and infectious diseases such as COVID-19 [37].

The paradigm changes from the reactive medicine to PPPM has been considered as a considerable transformation [5, 38]. Unlike the traditional manual interpretation mode, the AGS-based novel mode for COVID-19 screening and diagnosis can rapidly distinguish positive cases from mass samples and benefit to the subsequent adjustment and deployment of COVID-19 prevention and control measures under the PPPM framework. The diagnostic accuracy test indicates that the AGS with required precision is an optimal predictive and protective tool for COVID-19 to meet the “Chinese personalized COVID-free policy”. This study provides further strong supporting evidence for the application of PPPM in global COVID-19 epidemic control.

## Limitation

A limitation of this study should be noted. The rules of AGS in this study were designed to the application in Hong Kong and Shanghai. When AGS is applied in other environments, whether the setting of rules and parameters can meet the requirements of the local workstation remains to be further verified. The optimal parameters of the reference Ct value ranges for quality controls, positive and negative results would be calibrated and updated based on new information and real-world clinical scenario.

## Conclusions and expert recommendations

In conclusion, we created an AGS tool to automatically interpret the COVID-19 RT-PCR results and validated its efficiency in two mega cites, Hong Kong and Shanghai. Compared to conventional manual interpretation, the computer-based AGS not only shows a high accuracy but also considerably relieves both labour and time from the challenge of large-scale screening of SARS-CoV-2. This study supports the paradigm shift from reactive medicine to the proactive model of PPPM in the field of epidemiology of infectious diseases, demonstrating that the PPPM concept and strategy could be integrated into the diagnosis, prevention and intervention of global pandemic [39].

In recommendation, we provide (1) AGS as a PPPM tool which can be widely applied in clinical laboratories in the COVID-19 pandemic screening; (2) AGS would effectively facilitate the prediction of COVID-19 transmission risk by rapidly identifying and responding to the reported positive case at a real-world setting such as at densely populated cites, Hong Kong and Shanghai; (3) it also contributes to protect both the susceptive populations and infected patients with individual level of precision in the practice of PPPM; (4) AGS also has considerable time efficiency and cost effective to save the labour resource and meet the test-demanding in a real-word PPPM practice in the situation like COVID-19 pandemic.

## Data Availability

The dataset generated and analysed during the study are available from the corresponding author on reasonable request.

## Abbreviations

*AGS*: Autoverification and guidance system
*CLSI*: Clinical and Laboratory Standards Institute
*COVID-19*: Coronavirus disease 2019
*Ct*: Cycle threshold
*ELISA*: Enzyme-linked immunosorbent assay
*HBV*: Hepatitis B virus
*IgG*: Immunoglobulin G
*IgM*: Immunoglobulin M
*IM*: Individualized medicine
*N*: Nucleocapsid protein
*NAAT*: Nucleic acid amplification test
*NPV*: Negative predictive value
*ORF1ab*: Open reading frame1ab
*PCM*: Person-centred medicine
*PM*: Personalized medicine
*PPV*: Positive predictive value
*PPPM*: Predictive, preventive and personalized medicine
*Rt*: Effective reproduction number at time
*RT-PCR*: Reverse transcriptase-polymerase chain reaction
*SM*: Stratified medicine
*TATs*: Turnaround times
*TCAM*: Traditional, complementary and alternative medicine
*WHO*: World Health Organization
